# News from Societies

**Published:** 1956-10

**Authors:** 


### *News from Societies*

### PROGRAMMES 1956–57 SESSION

### BRISTOL MEDICO-CHIRURGICAL SOCIETY

On 10th October Dr. E. G. Brabbeer, F.F.A.R.C.S., was installed as President for the current session and gave an address on "Advances in Anaesthesia in the last thirty years".

Professor C. Bruce Perry, M.D., F.R.C.P., was elected President for the Session 1957–58.

14th Nov.: **Clinical Meeting** at Southmead Hospital.

12th Dec.: Sir Horace Evans, K.C.V.O., F.R.C.P., **"Hypertension and Artery Disease".**

9th Jan.: Professor C. Bruce Perry, M.D., F.R.C.P., **"Prevention of Acute Rheumatism".**

13th Feb.: Professor T. F. Hewer, M.D., F.R.C.P., and others, **"Clinico-Pathological Conference".**

13th Mar.: Mr. G. L. Alexander, F.R.C.S., **"Head Injuries".**

10th Apr.: Dr. J. H. Middlemiss, M.D., F.F.R., D.M.R.D., **"Diagnostic Radiology".**

8th May: Professor Sir Robert MacIntosh, D.M., F.R.C.S. (Edin.), F.F.A.R.C.S., **"Two months behind the Iron Curtain".**

### B.M.A. (Bristol Division)

24th Oct., 8.30 p.m.: Professor G. Gordon Lennon, Professor of Obstetrics and Gynaecology, Southmead Hospital, **Film—Turkey and Iraq.** Senior Common Room, University of Bristol.

2nd Nov., 8 p.m.–1 a.m.: **Dance.**—Grand Spa Hotel, Clifton, Bristol 8.

23rd Jan., 1957, 8.30 p.m.: **Visit to New Council House, College Green, Bristol.** The Lord Mayor and Lady Mayoress will receive members and wives.

20th Mar., 8.30 p.m.: Dr. Doris Odlum, Consultant Psychiatrist, **"Medical Aspects of Homosexuality".** Main Physics Lecture Theatre, Royal Fort.

15th May, 8.30 p.m.: **Annual General Meeting.** Main Physics Lecture Theatre, Royal Fort.

*Summer, 1957: **Social Event**—to be arranged.

*21st June, 4 p.m.: B.M.A., **Reception for newly-qualified doctors** (Bristol University), Garden Hall, Royal Fort, Bristol.

26th June, 8.30 p.m.: **Clinical Meeting** at Bristol General Hospital.

* Provisional dates.

### WEST OF ENGLAND CHILD HEALTH GROUP

12th Oct., 8.30 p.m.: A. V. Neale, Esq., M.D., F.R.C.P., D.P.H., Professor of Child Health, University of Bristol, **"Paediatrics in the Caribbean".** Department of Child Health Lecture Theatre, Children's Hospital.

8th Nov., 8.30 p.m.: J. F. Coates, Esq., M.D., M.R.C.P., Consultant Paediatrician, Musgrove Park Hospital, Taunton, **"The Child with Respiratory Allergy".** Department of Child Health Lecture Theatre, Children's Hospital.

7th Dec., 8.30 p.m.: Dr. Alice Stewart, M.A., M.D., F.R.C.P., Reader in Social Medicine, University of Oxford, **"Research into Child Health by a Department of Social Medicine".** Department of Child Health Lecture Theatre, Children's Hospital.

17th Jan., 5.30 p.m.: D. E. Milner, Esq., O.B.E., A.R.C.A., R.W.A., Principal, West of England College of Art, **"Child Art".** Department of Child Health Lecture Theatre, Children's Hospital.

21st Feb., 5.30 p.m.: J. C. Spencer, Esq., M.A., PH.D., Research Fellow in Social Studies, University of Bristol, **"Neighbourhood and Family".** Department of Child Health Lecture Theatre, Children's Hospital.

28th Mar., 8.30 p.m.: R. Lightwood, Esq., M.D., F.R.C.P., D.P.H., Director, Paediatric Unit, St. Mary's Hospital Medical School, Physician, Hospital for Sick Children, Great Ormond Street, **"The Home Care of Sick Children".** Department of Child Health Lecture Theatre, Children's Hospital.

April: Members of the Staff of the Children's Hospital, **Clinical Meeting.** Out-Patient Department, Children's Hospital.

25th May, 2.30 p.m.: **Field Visit.** Croydon Hall Special School, Watchet, Somerset.

### B.M.A. (Gloucestershire Branch)

11th Oct. at Gloucester: W. J. Wilkin, Esq., F.R.C.S., Presidential Address, **"The Problem of the Varix".**

8th Nov. at Cheltenham: **Clinical Meeting.**

6th Dec. at Gloucester: Mr. G. N. Pattison, **Paper and illustrative films on an ophthalmic subject.**

10th Jan. at Cheltenham: **Symposium on research in General Practice.**

14th Feb. at Gloucester: S. A. Bond, Esq., F.R.C.S., **Paper on gynaecological subject.**

14th Mar. at Cheltenham: To be arranged.

11th April at Gloucester: **Clinical Meeting.**

9th May at Stroud: R. Merryweather, Esq., F.R.C.S., **Annual General Meeting and paper on orthopaedic subject.**

13th June at Gloucester: **Address by the President's Guest.**

The Gloucester meetings will be held at the Wheatstone Hall, Brunswick Road, Gloucester, with the exception of the clinical meeting in April which will be held at the Gloucestershire Royal Infirmary. The Cheltenham meetings will be held at Cheltenham General Hospital.

### B.M.A. (West Somerset Division)

27th Oct.: **Clinical Demonstration on Cardiovascular Diseases** in Musgrove Park Hospital, Taunton.

22nd Nov.: **Annual Dinner and Dance** in the Empire Hall, County Hotel, Taunton.

6th Dec.: **Clinical Meeting** in Yeovil Hospital, Yeovil, when Dr. J. G. Howells, Department of Child Psychiatry, Ipswich, will speak on "Family Psychiatry and the G.P."

Early in April, 1957: **Dining Meeting.**

### B.M.A. (Barnstaple Division)

13th Oct.: **Social evening.** Alexandra Hospital, Barnstaple.

14th Nov.: B. Moore, Esq., B.SC., M.D., **"Virus Diseases and the Virus Disease Laboratory."** N. Devon Infirmary, Barnstaple.

### COSSHAM MEDICAL SOCIETY

Meetings held at Cossham Hospital, coffee at 8.45 for 9 p.m. meeting.

2nd Oct.: Dr. G. I. Culank, M.R.C.S., L.R.C.P., **"Presidential Address".**

6th Nov.: G. I. Strachan, Esq., C.B.E., HON. LL.D., M.D., F.R.C.P., F.R.C.S., F.R.C.O.G., Emeritus Professor University of Wales, **"Gynaecological Problems in General Practice".**

4th Dec.: Professor G. G. Lennon, CH.M., F.R.C.O.G., M.M.S.A., and R. E. Hemphill, M.A., M.D., D.P.M., **"Brains Trust".**

8th Jan., 1957: G. D. Roberts, Esq., Q.C., Recorder of Bristol, **"Doctor in the Witness Box".**

5th Feb.: Frank Bodman, Esq., M.D., **"Homeopathy".**

5th Mar.: Surgeon Commander W. A. Burnett, R.N., **"Modern Methods of Submarine Escape".**

2nd April: **Annual General Meeting.**

### CLINICAL SOCIETY OF BATH

Meetings take place at the Royal United Hospital on the second Friday in the month at 8 p.m. Cases are shown at 8, briefly discussed at 8.30, and this is followed by the paper of the evening.

12th Oct.: Conjoint meeting with College of General Practitioners. A clinical evening.

9th Nov.: Mr. F. D. Murphy, **"Some Aspects of Peptic Ulcer".**

14th Dec.: Dr. Tovey, of South Western Region Blood Bank. (Title to be announced later.)

11th Jan., 1957: Mr. Hedley Hall, **"Scoliosis".**

8th Feb.: Dr. C. Hagenbach, **"Migraine".**

8th Mar.: **Clinical Conference.** (Details to be considered nearer the date.)

12th April: Dr. Ritchie Russell, of Oxford, **"Poliomyelitis".**

### WEST PENWITH MEDICAL SOCIETY

The oldest medical society in Cornwall. Holds its meetings, unless otherwise stated, at 8.30 p.m., on the third Thursday in the month, at the West Cornwall Hospital, Penzance.

Visitors are welcomed, and should make themselves known to the Secretary, Dr. Douglas Truscott. There are no meetings in August.

1956

Sept.: Dr. S. A. Greenwood-Penny, Dr. D. R. Murley, **"Ancient Cornish Remedies".**

Oct.: **Annual Dinner.**

Nov.: H. W. Hughes, Esq., M.D., M.R.C.P., Medical Superintendent, Tehidy Sanatorium, Camborne, **"The Tuberculous Child".**

14th Dec.: Dr. D. H. Davies, Physician and Cardiologist, Bristol Royal Infirmary, **"Hypertension".**

1957

18th Jan.: Dr. J. H. Middlemiss, M.D., F.F.R., Director, Radiodiagnostic Department, Bristol Royal Infirmary. Title to be announced.

Feb.: Dr. Johnstone, D.P.H., Medical Officer of Health, West Penwith. Title to be announced.

March:

March: Dr. L. W. Hale, F.R.C.P., Consultant Physician, Redruth Miners and General Hospital. Title to be announced.

April: No Meeting. The Society combines with the B.M.A. for the B.M.A. Clinical Lecture.

May: Dr. A. M. Brunton, Deputy Director, Medical Services, Pfizer Ltd., **"Recent Work on Corticosteroids".**

June: Dr. Walker, PH.D., Physicist, Radiological Protection Service, **"Physics in Medicine".**

July: Dr. Philip Bryd, Chief Medical Officer, Associated Ethyl Co.. Title to be announced.

### SOUTH SOMERSET MEDICAL CLUB

18th Oct.: **A.G.M. and Film Show.**

15th Nov.: Dr. Hugh Jolly, **"Paediatrics in General Practice".**

17th Jan.: **Annual Dinner and Dance.**

21st Feb.: Dr. Macdonald Critchley, **"The Romance of Huntingdon's Chorea".**

18th April: To be arranged.

### PLYMOUTH MEDICAL SOCIETY

26th Sept., at 2.30 p.m.: **Joint Meeting with B.M.A.** Medical Rehabilitation Unit, R.A.F. Collaton Cross, by courtesy of the Commanding Officer, Group-Captain H. L. Willcox.

5th Oct., at 8.30 p.m.: Lawrence Abel, Esq., M.S., F.R.C.S., B.M.A. Lecture, **"Common Diseases of the Rectum and Anal Canal".** Freedom Fields.

19th Oct., at 8.30 p.m.: Short papers by Robert Blair, Esq., M.D., D.P.M., Paul Bramley, Esq., M.B., F.D.S., John Dawson, Esq., M.D., M.R.C.P., Ian Payne, Esq., M.B., F.R.C.S., D.O.M.S., Michael Reilly, Esq., M.S., F.R.C.S., Francis Wheeldon, Esq., M.B., F.R.C.S. Freedom Fields.

24th Oct.: **Annual Dinner and Dance.** The Dartmoor Restaurant (E. Dingle & Co.), Royal Parade.

9th Nov., at 8.30 p.m.: **Clinical Evening.** Greenbank.

23rd Nov., at 8.30 p.m.: Professor G. J. Cunningham, M.B.E., M.D., Professor of Pathology, Royal College of Surgeons. B.M.A. Lecture, **"Interesting Giants and Dwarfs in the last few Centuries".** Freedom Fields.

7th Dec., at 8.30 p.m.: **Clinical Evening.** Greenbank.

18th Jan., at 8.30 p.m.: Dr. J. H. Sheedon, F.R.C.P., **"Problems of Old Age".** Freedom Fields.

1st Feb., at 8 p.m.: **Clinical Evening** at the Royal Naval Hospital, by courtesy of Surgeon Rear-Admiral A. A. Pomfret, O.B.E., Q.H.S., R.N.

15th Feb., at 8.30 p.m.: A. Dickson Wright, Esq., M.S., F.R.C.S., **"How Patients have deceived me".** Freedom Fields.

1st Mar., at 8.30 p.m.: Professor E. P. Sharpey-Schafer, F.R.C.P. Freedom Fields.

13th Mar., at 2.30 p.m.: Visit to Dame Hannah Rogers School for Cerebral Palsy, Ivybridge.

### THE DEVON AND EXETER MEDICO-CHIRURGICAL SOCIETY

4th Oct., at 8.15 p.m.: Mr. Dendy Moore, F.R.C.S., **"The use of the radio-opaque medium Diodone in surgical diagnosis and treatment".**

18th Oct., at 4 p.m.: Dr. J. W. Cook, F.R.S., Vice-Chancellor of Exeter University, **"Chemical Carcinogens and their Significance".** (South Western Dinner.)

15th Nov.: **Clinical Meeting.**

3 p.m.: Cases on view in Medical Out-Patients Department.

4 p.m.: Tea, followed by Discussion on Cases in Library.

13th Dec.: The President's Party to which wives of members are invited.

8 p.m.: The Board Room.

9 p.m.: The Library. (The Elizabethan Singers.)

17th Jan., at 8.15 p.m.: J. Lloyd Griffith, Esq., F.R.C.S., G. F. Trobridge, Esq., M.B., M.R.C.P., Anthony Daly, Esq., M.D., M.R.C.P., **Symposium on The Surgery of the Heart.**

21st Feb., at 8.15 p.m.: **Clinico-Pathological Demonstration.**

21st Mar.: **Clinical Meeting.**

3 p.m.: Cases on view in Medical Out-Patients Department.

4 p.m.: Tea, followed by Discussion on Cases in Library.

4th April, at 3 p.m.: **Clinical Meeting** at the Princess Elizabeth Orthopaedic Hospital, followed by tea.

25th April, at 8.15 p.m.: **Annual General Meeting, Clinico-Pathological Demonstration.**

### TORQUAY AND DISTRICT MEDICAL SOCIETY

All meetings are on Thursdays, and at Torbay Hospital unless otherwise stated.

25th Oct., at 4 p.m.: Opening Meeting, Sir Charles Symonds, K.B.E., C.B., M.D., F.R.C.P., **"Vertigo".**

7.45 for 8.15 p.m.: Dinner and Dance at the Palace Hotel, Torquay.

8th Nov., at 8.30 p.m.: Dr. W. A. Gillespie, **"Modern Antibiotics".**

22nd Nov., at 3 p.m.: Combined Clinical Meeting with the S.W. Branch of the British Medical Association.

13th Dec., at 8.30 p.m.: Sir Heneage Ogilvie, K.B.E., M.D., M.CH., F.R.C.S., **"Medical Rackets".**

24th Jan., at 8.30 p.m.: Mr. John Peel, F.R.C.S., F.R.C.O.G., **"Some Common Gynaecological Problems in Everyday Practice".**

14th Feb., at 8.30 p.m.: Combined Clinical Discussion with the Clergy, **"Abuse of Sedation".** Victoria Hotel, Torquay.

21st Mar., at 8.30 p.m.: Mr. Lawrence Abel, F.R.C.S., **"Common Diseases of the Rectum".**

25th April, at 8.30 p.m.: Dr. Peter Bishop, M.R.C.P., **"Facts and Fancies in Clinical Endocrinology".**

30th May, at 8.30 p.m.: **Annual General Meeting.**

### CORNWALL CLINICAL SOCIETY

The pattern of the evenings will be the same as before: refreshments are served from 8.0 to 8.30 and the Meeting takes place from 8.30, finishing not later than 10.30.

1st Nov.: (1) Dr. C. J. F. Coombs, **"Birds and Men: Their Behaviour and Some Comparisons"**; (2) Dr. C. T. Andrews, **"The Romance of John Keats, Apothecary and Poet."** (Illustrated by lantern slides). Royal Cornwall Infirmary (Ladies' Night).

29th Nov.: **B.M.A. Dinner and Dance.**

7th Dec.: **Clinical Evening.** Camborne–Redruth Hospital.

10th Jan.: **"Any Questions".** A Medical Quiz with a Specialist Panel. Royal Cornwall Infirmary.

1st Feb.: Dr. Hugh Jolly, **"Paediatrics in Modern General Practice".** Camborne–Redruth Hospital.

7th March: **Clinical Evening.** Royal Cornwall Infirmary.

April: Sir Heneage Ogilvie, **"Diagnosis of the Jaundiced Patient".** Royal Cornwall Infirmary.

2nd May: Various Contributors, **"Personal Experiences of Illnesses and Operations".** Camborne–Redruth Hospital.

7th June: **Clinical Evening.** St. Lawrence's Hospital, Bodmin.

4th July: **Cases and Demonstrations by the Consultant Staff.** Royal Cornwall Infirmary.

3rd Oct.: **Presidential Address and Annual General Meeting.** Royal Cornwall Infirmary.

### POST-GRADUATE COURSES

### UNIVERSITY OF BRISTOL

The following courses are being arranged:

Week-end Course at Plymouth, 30th Nov. to 2nd Dec.

Ward-rounds at Southmead Hospital, Dec. 1956 to March 1957.

Dental Course, 8th to 13th April 1957.

Sunday morning Lecture-Demonstrations, April to June 1957.

Further details may be obtained from the Department of Post-Graduate Medical Studies.

### ROYAL DEVON AND EXETER HOSPITAL

Meetings will be held at 8.30 p.m. on Tuesdays in the Library.

16th Oct.: Mr. J. E. Richardson, **"Stomachs in East London".**
23rd Oct.: Dr. N. S. Alcock, **"Neurological Emergencies".**
30th Oct.: Mr. C. C. Jeffery, **"Treatment of Fractures in the Aged".**
6th Nov.: Dr. H. R. Jolly, **"Paediatrics in Modern General Practice".**
13th Nov.: Dr. G. D. Adkins, **"Chemotherapy of Tuberculosis".**
20th Nov.: Mr. A. C. Gairdner, **"Urinary Obstruction".**
27th Nov.: Mr. G. N. Cantrell, **"Some Ophthalmic Problems".**
4th Dec.: Dr. A. J. Daly, **"Salt and Water".**
11th Dec.: Dr. M. G. Thorne, **"Uses and Dangers of Steroid Therapy".**
18th Dec.: Dr. C. M. Seward, Mr. H. D. Moore, Dr. J. O. P. Edgcumbe, Mr. D. Jefferiss, Dr. F. S. W. Brimblecombe, Dr. N. Sawers, Chairman: Dr. A. J. Daly, **Panel Discussion: "Any Questions".**

